# Body-Worn Sensors for Remote Monitoring of Parkinson’s Disease Motor Symptoms: Vision, State of the Art, and Challenges Ahead

**DOI:** 10.3233/JPD-202471

**Published:** 2021-07-16

**Authors:** Silvia Del Din, Cameron Kirk, Alison J. Yarnall, Lynn Rochester, Jeffrey M. Hausdorff

**Affiliations:** a Translational and Clinical Research Institute, Faculty of Medical Sciences, Newcastle University, Newcastle upon Tyne, UK; b Newcastle upon Tyne Hospitals NHS Foundation Trust, Newcastle upon Tyne, UK; c Center for the Study of Movement, Cognition and Mobility, Neurological Institute, Tel Aviv Sourasky Medical Center, Tel Aviv Israel; d Department of Physical Therapy, Sackler School of Medicine and Sagol School of Neuroscience, Tel Aviv University, Tel Aviv, Israel; e Rush Alzheimer’s Disease Center and Department of Orthopaedic Surgery, Rush University Medical Center, Chicago, IL, USA

**Keywords:** Parkinson’s disease, remote monitoring, real-world, wearables, motor symptoms, accelerometer

## Abstract

The increasing prevalence of neurodegenerative conditions such as Parkinson’s disease (PD) and related mobility issues places a serious burden on healthcare systems. The COVID-19 pandemic has reinforced the urgent need for better tools to manage chronic conditions remotely, as regular access to clinics may be problematic. Digital health technology in the form of remote monitoring with body-worn sensors offers significant opportunities for transforming research and revolutionizing the clinical management of PD. Significant efforts are being invested in the development and validation of digital outcomes to support diagnosis and track motor and mobility impairments “off-line”. Imagine being able to remotely assess your patient, understand how well they are functioning, evaluate the impact of any recent medication/intervention, and identify the need for urgent follow-up before overt, irreparable change takes place? This could offer new pragmatic solutions for personalized care and clinical research. So the question remains: how close are we to achieving this? Here, we describe the state-of-the-art based on representative papers published between 2017 and 2020. We focus on remote (i.e., real-world, daily-living) monitoring of PD using body-worn sensors (e.g., accelerometers, inertial measurement units) for assessing motor symptoms and their complications. Despite the tremendous potential, existing challenges exist (e.g., validity, regulatory) that are preventing the widespread clinical adoption of body-worn sensors as a digital outcome. We propose a roadmap with clear recommendations for addressing these challenges and future directions to bring us closer to the implementation and widespread adoption of this important way of improving the clinical care, evaluation, and monitoring of PD.

## INTRODUCTION: THE “VISION”—ARE WE THERE YET?

At the 2013 World Congress of the International Society of Posture and Gait Research, a keynote speaker predicted that digital health technology such as body-worn sensors (BWS) would soon become a routine, widely used tool to augment the clinical examination of patients with Parkinson’s disease (PD) and, more importantly, enhance patients’ quality of life [1]. Accumulating evidence at that time demonstrated that low-cost, easy-to-use BWS could be used in the clinic to provide new information and add needed objectivity to the assessment of PD motor symptoms, gait, and mobility. Use of BWS could, therefore, help patients by enhancing their therapeutic management, function and quality of life, and offer them a personalized approach to their symptoms. Moreover, an emerging, exciting set of studies demonstrated that continuous, “24/7” remote monitoring empowered by BWS had the potential to measure, characterize, and quantify both subtle and large changes in mobility and other critical motor symptoms of a patient with PD, providing a robust comprehensive map of the patient’s function and its changes over time. In this vision, a patient would receive a small package in the mail containing a BWS several weeks before a routine clinical exam. After following simple instructions and wearing the device continuously for one week, the information collected would be uploaded to a cloud, automatically analyzed, summarized, sent to the clinician for review and provide feedback to the patient. At the clinical exam, the healthcare professional would read an objective, detailed report on the patient’s motor function that could be compared and contrasted to the results of previous years, even before the patient took a single step into the clinic. In this way, the clinical visit would be transformed into a more productive and informed meeting, enhancing clinical care in a truly personalized manner.

This optimistic vision has not yet been realized [2, 3]. Nonetheless, at a time when COVID-19 is wreaking havoc throughout the globe, the need has become even greater. In this short review, we provide an overview of the current use of BWS (accelerometers, inertial measurement units (IMUs)) for the remote monitoring of PD motor symptoms, summarize the challenges that must be overcome to achieve that potential, and outline steps that should and are being taken, in the long road ahead [2] to address this important opportunity for improving the evaluation and monitoring of PD motor symptoms.

## STATE OF THE ART: WHERE ARE WE AT?

The rapidly expanding field of remote monitoring has been the subject of several recent systematic reviews [4–7]. Here, we provide an overview of recent representative work (2017–2020) on remote monitoring of PD motor symptoms using BWS (Table 1). We report studies using BWS for remote monitoring (real-world, at-home and in the community) in both unsupervised or scripted conditions, in order to quantify digital outcomes, focusing on papers related to motor symptoms and motor complications: tremor, bradykinesia, dyskinesia, postural instability, gait disturbances and turning, falls risk, freezing of gait (FoG) and physical activity. Using a previously proposed framework [8], in Table 1 we report:

**Table 1:** Representative studies examining remote monitoring of Parkinson’s disease (PD) motor symptoms using body worn sensors (BWS), between 2017 and 2020. Validation of digital outcomes has been classified ‘yes’ for each of the following criteria: 1. criterion validity: if digital outcome has been validated against a gold standard reference in the study cited, or in previous studies; 2. construct validity: if digital outcome has been validated (e.g., correlated) against clinical scales (convergent validity) and/or it has shown significant differences between groups (discriminant validity) in the study cited, or in previous studies. Digital outcome regulated/qualified has been classified ‘yes’ if BWS and/or digital outcome has received FDA (510K^1^) or EMA^2^ positive decision/qualification

Study, Year	Dataset	Protocol	BWS Type/Position	Clinical Concept of Interest	Digital Outcome	Digital Outcome Validated (1. criterion validity, 2. construct validity)	BWS and/ or Digital Outcome Regulated/ Qualified
*Tremor, bradykinesia, dyskinesia, motor fluctuations*							
Samà, et al., 2017 [13]	12 PD	1 day^*^ (40 min), scripted tests ON and OFF state	IMUs (9 x 2)/Waist	Bradykinesia	Gait, frequency domain features for SVM model	1. Yes, at home (SVM, against videos)	No
						2. Yes (convergent validity (UPDRS, UPDRS-III))
Tsiouris et al., 2017 [14]	20 PD	Scripted tests	PD manager: IMUs (Microsoft Band), Sensor insoles (Moticon), Smart Pillbox (SimpleMed+, Vaica), Smartphone/Wrist, Feet	Tremor, dyskinesia, bradykinesia, gait, FoG	Amplitude and constancy of tremor/not detailed features for ML techniques	1. No 2. Yes (convergent validity (UPDRS))	No
Wagner et al., 2017 [17]	19 PD	2 days	Accelerometer (GENEActiv)/Wrist	Tremor, bradykinesia, dyskinesia	Wavelet features (contribution and relative energy of each scale)	1. Yes, in the lab (SVM, against clinician scores)	No
						2. No
Farzanehfar et al., 2018 [12]	103 PD	6-7 days, unsupervised	Accelerometer (PKG, Global Kinetic Corporation)/Wrist	Bradykinesia, dyskinesia	Bradykinesia score classified as movements with lower acceleration and amplitude. Dyskinesia classified as movements of normal amplitude and acceleration, but shorter periods without movement	1. Yes, previous work [49] 2. Yes, (convergent validity (UPDRS III))	Yes
Rodríguez-Molinero et al., 2018 [15]	23 PD	1–3 days	IMUs (9×2)/Waist	Bradykinetic gait, dyskinesia, ON-OFF state	Bradykinesia fluidity measure (frequency domain measure, power spectra 1–10Hz for each stride), dyskinesia (power spectra 1–4Hz for each stride)	1. Yes (SVM, against diaries) 2. No	No
Rodríguez- Molinero, et al., 2019 [16]	13 PD	30 min of scripted activities	IMUs (9×2)/Waist	Dyskinesia	Power spectrum density in the frequency band comprised of harmonics of 1–4Hz	1. Yes, in home environment using video data	No
						2. Yes, concurrent validity against clinical scales
Coates, et al., 2020 [46]	5 PD, 5 OA	7 days, unsupervised	Axivity AX3/Lower back	Motor symptom severity (MDS-UPDRS III)	Sample entropy (SampEnt)	1. No	No
						2. Yes (convergent validity (against UPDRS III &levodopa equivalent daily dose (LEDD)) and discriminant validity (PD vs. CL))
Evers et al., 2020 [11]	25 PD, 25 CL	1 day, scripted tests at home	IMUs (Gait Up Physilog 4, Android Wear smartwatch), contextual (smartphone) and physiological (Empatica E4) sensors/Lower back, wrists, ankles, pocket	ON-OFF state, FoG	Gait: Frequency domain measures (power spectral density (PSD), total power in the 0.5-10 Hz band, frequency, height and width of PSD dominant frequency)	1. No 2. Yes (convergent validity (ON vs. OFF state) and discriminant validity (PD vs. CL))	No
*Postural instability, gait disturbances, and turning*							
Rodríguez-Molinero et al., 2017 [26]	75 PD	1 day^*^, clinical assessment and scripted tests at home in ON and OFF state	IMUs (9×3)/Waist	UPDRS-III (axial function, balance, and gait)	Scalar value for ON-OFF state based on frequency domain features (power spectra 1–10Hz for each stride).	1. Yes, in the lab, previous work, SVM against videos [50] 2. Yes (convergent validity (UPDRS-III, UPDRS-III factor 1: “axial function, balance, and gait.”))	No
Haertner et al., 2018 [27]	55 PD	12 days (median)	IMUs (RehaGait^®^, Hasomed)/Lower back	Turning, falls risk	Duration, angle, average angular velocity, starting, middle and ending angular velocity and maximum angular velocity	1. Yes, in home-like environment, previous work [51]	No
						2. Yes (discriminant validity (various PD fallers types))
Mancini et al., 2018 [25]	94 PD (25 freezers)	3 days, unsupervised, clinical assessment and scripted test at home	IMUs (Dynaport Hybrid, McRoberts)/Lower back	Turning, FoG	Mean and coefficient of variation (CV) of: number of turns per 30 min, turn angle amplitude, turn duration, mean and peak turn velocity, turn jerkiness, turn medio-lateral range of acceleration.	1. Yes, in the lab for turning, previous work [52] 2. Yes (convergent validity (NFOG-Q) and discriminant validity (freezers vs. non-freezers)).	No
Shah et al., 2020 [22]	29 PD, 20 OA	7 days, unsupervised^*^	IMUs (Opal, APDM)/Lower back, Feet	Gait	Gait speed, stride length, cadence, double-support, swing duration, pitch of feet at initial ground contact, frequency of bout length (number of strides) over a week	1. Yes, in the lab, previous work [53]	No
						2. Yes (convergent validity (UPDRS-III, PIGD, previous work) and discriminant validity (PD vs. OA))
Shah et al., 2020 [24]	29 PD, 27 CL	7 days, unsupervised^*^	IMUs (Opal, APDM)/Lower back, Feet	Gait, turning	43 digital mobility characteristics (lower body, upper body, turning, activity, variability)	1. Yes, in the lab, previous work [52, 53] 2. Yes (discriminant validity (PD vs. CL))	No
Shah et al., 2020 [23]	29 PD, 20 CL, 13 MS, 21 CL	7 days, unsupervised^*^	IMUs (Opal, APDM)/Lower back, Feet	Gait, turning	46 digital mobility characteristics (lower body, upper body, turning, activity, variability)	1. Yes, in the lab, previous work [52, 53]	No
						2. Yes (convergent validity (UPDRS-III, PIGD) and discriminant validity (PD vs. CL))
*Falls risk, freezing of gait (FoG)*							
Rodríguez-Martín et al., 2017 [36]	21 PD	1 day^*^ (40 mins), scripted tests ON and OFF state	IMUs (9×2)/Waist	FoG	55 features for real-time SVM model	1. Yes, at home (against videos) 2. No	No
Rodríguez-Martín et al., 2017 [37]	12 PD	3 days^*^, unsupervised, and in-lab scripted tests	IMUs (9×3)/Waist	FoG, bradykinetic gait	55 features for real-time SVM model, frequency domain measures (strides)	1. Yes, at home (against videos) 2. No	No
Mancini et al., 2018 [54]	24 PD	7 days^*^, unsupervised, clinical assessment and scripted test at home	IMUs (Opal, APDM)/Lower back, Ankles	FoG	Average of time spent freezing per hour (Total % time with Freezing ratio > 1 normalised on recording time), variability of % time spent freezing, turning and walking features	1. Yes, in the lab, for turning, previous work [52] No for FoG and walking	No
						2. Yes for FoG, turning, and walking (convergent validity (NFOG-Q and ABC) and known group differences (freezers vs. non-freezers)).
Del Din et al., 2019 [29]	155 PD F, 122 OA F, 15 PD NF, 50 OA NF	7 days, unsupervised	Accelerometer, (AX3, Axivity)/Lower back	Falls risk, ambulatory activity	14 Micro gait characteristics (pace, rhythm, variability, asymmetry, postural control), 7 Macro gait characteristics (volume, pattern, variability)	1. Yes, in the laboratory for Micro gait characteristics, previous work [55]. In real-world for Macro gait characteristics in YA, previous work [56]	No
						2. Yes (convergent validity (FES-I), and discriminant validity (PD vs. OA, F vs. NF))
Del Din et al., 2020 [30]	128 PD F,109 OA F,38 MCI F	7 days, unsupervised	Accelerometer, (AX3, Axivity)/Lower back	Falls risk, ambulatory activity	7 Macro gait characteristics (volume, pattern, variability), fall rates relative to activity exposure (FRA) index	1. Yes, in real-world for Macro gait characteristics in YA, previous work [56]	No
						2. Yes (convergent validity (FES-I), previous work, and discriminant validity (PD vs. OA))
Reches et al., 2020 [38]	71 PD	FoG provoking test in the lab in ON and OFF states	IMUs (Opal, APDM)/Lower back, Feet	FoG	86 features from previous work for SVM model.	1. Yes, in the lab against labelled video	No
						2. Yes (convergent validity (NFOG, UPDRS-III and TUG time), discriminant validity (OFF vs. ON state))
Sigcha et al., 2020 [57]	21 PD	20 minutes at home, scripted ADLs in ON and OFF states	IMUs (9×2)/Waist	FoG	Mean, standard deviation, variance, frequency, entropy, energy, freeze index, sum of freeze index, locomotion band and variables related to FFT. ML and DL models from previous work.	1. Yes, at-home against labelled video 2. No	No
*Physical activity*							
Cai et al., 2017 [42]	21 PD, 20 CL	5 days, unsupervised	Bong Smart Sports bracelet/Wrist	Physical activity	Average daily physical activity amount and calories	1. Yes, using self-report diaries 2. Yes (convergent validity (UPDRS –III, H&Y, Levodopa) and discriminant validity (PD vs. CL))	No
Silva de Lima et al., 2018 [41]	304 PD	13 weeks	Pebble watch/Wrist	Physical activity/motor fluctuations (ON/OFF state)	Mean time spent walking	1. No	No
						2. Yes (convergent validity (UPDRS item 4.4))
Galperin, et al., 2019 [43]	125 PD	7 days, unsupervised	Accelerometer, Axivity (AX3)/Lower back	Physical activity	Acceleration derived features: Number of steps, number of walking bouts, step length, step regularity, amplitude of dominant frequency, SD of the peaks amplitude CV) and signal vector magnitude (SVM)	1. Yes, in the laboratory against another BWS (GENEActiv which has been validated in previous work [58] 2. Yes (convergent validity (UPDRS-III))	No
Pradhan et al., 2019 [40]	30 PD, 30 OA	14 days, unsupervised	Fitbit Charge HR/Wrist	Physical activity	Daily step count and METs	1. Yes, in the laboratory and outdoor, previous work [59]	Yes (only ECG App)
						2. No
Ito et al., 2020 [44]	13 PD	1–7 days^*^	Accelerometer (Active Style Pro HJA 750C, OMRON)/Waist	Physical activity and motor symptoms (ON state, dyskinesia)	MET, PAL	1. Yes in the laboratory, previous work [60]	No
						2. Yes (convergent validity (UPDRS-III, ON state, dyskinesia))

^*^Night excluded. ABC, Activity specific Balance Confidence scale; ADLs, Activities of Daily Living; CL, Controls; DL, Deep Learning; ECG, Electrocardiogram; F, Fallers; FES, Falls Efficacy Scale; H&Y, Hoehn and Yahr; MET, Metabolic Equivalent; ML, Machine Learning; MS, People with Multiple Sclerosis; NF, Non-fallers; NFOG-Q, New freezing of gait questionnaire; MDS-UPDRS, Movement Disorder Society Unified Parkinson’s disease Rating Scale; OA, Older Adults; PAL, Physical Activity Level; PD, People with Parkinson’s disease; PKG, Parkinson’s KinetiGraph; SVM, Super Vector Machine; TUG, Timed Up and Go; UPDRS-III, Unified Parkinson’s disease Rating Scale, Part III. ^1^https://www.accessdata.fda.gov/scripts/cdrh/cfdocs/cfpmn/pmn.cfm. ^2^https://www.ema.europa.eu/en/human-regulatory/research-development/scientific-advice-protocol-assistance/novel-methodologies-biomarkers/opinions-letters-support-qualification-novel-methodologies-medicine-development

(a) the clinical concept of interest;
(b) quantified digital outcomes;
(c) validity:
  i. criterion validity: digital outcome validated against a reference system,
  ii. construct validity: digital outcome validated against clinical scales (convergent validity) and/or showed known groups differences (discriminant validity));
(d) digital outcome regulatory/qualification status assessed by regulatory bodies (e.g., EMA, FDA) [9, 10].

### Tremor, bradykinesia, dyskinesia, motor fluctuations

BWS have been used to automatically detect and evaluate tremor, bradykinesia, dyskinesia and on/off medication state [11–17]. The main techniques for identification of these symptoms are based on machine learning (ML) models (e.g., support vector machines (SVM)). Models are usually fed with digital outcomes that are signal-based features (e.g., frequency domain) extracted from the BWS.

In terms of criterion validity, studies tend to validate the digital outcome against a reference system (e.g., videos, self-report), using mainly ML techniques, showing good accuracy (>90%) [13, 15, 17]. Construct validity is generally tested utilizing clinical scales (e.g., Unified Parkinson’s disease Rating Scale (UPDRS)), but is less explored.

Although preliminary results are promising for some digital outcomes, excluding Farzanehfar et al. [12], these studies include a limited number of subjects (≤25) and, therefore, the generalizability of ML models and related validity for clinical adoption is problematic. We note using BWS to study rigidity, one of the cardinal symptoms of PD, is especially challenging [18].

### Postural instability, gait disturbances, and turning

The importance of postural instability, gait, and turning as diagnostic, prognostic, and progression markers in PD is well recognized [19–21]. Nonetheless, static balance tests are usually confined to laboratory environments. Difficulty in identifying and discriminating periods of static, “quiet” standing balance from sedentary behavior during everyday activities (especially using single BWS on the trunk) and achieving a “totally unsupervised” postural instability assessment is challenging.

Evaluations of digital outcomes are based on: first identification in the BWS signal of the clinical concept of interest (e.g., gait, turning) using either ML methods or previously validated signal-based methods (e.g., methods developed from lab-based validation against gold standards); and second on the quantification of digital outcomes in the identified segments of the signals. Digital outcomes often include signal-based features (e.g., extracted from the BWS signal – frequency domain) or clinically relevant and “translatable” features (e.g., walking speed).

Recent studies on the construct validity of turning and gait corroborated that real-world gait and turning performances of PD were impaired (e.g., slower, more variable, and with lower cadence), compared with older adults [22–24] and reported moderate correlations with clinical scales (e.g., UPDRS) [25, 26]. Only a few studies have reported criterion validity for digital outcomes, and this was limited to in laboratory or home-like environments [25, 27, 28]. Real-world validation remains challenging and relies mainly on videos as a reference.

### Falls risk, freezing of gait

BWS can help advance our understanding of fall risk. For these concepts of interest, the main techniques are again use of ML models to identify relevant segments of the BWS signals, and then quantification of digital outcomes by using signal-based features or validated clinically relevant digital outcomes (e.g., walking speed, variability).

Results show that quality (micro) and quantity (macro) digital outcomes describing gait and turning are associated not only with falls status (fallers vs. non-fallers) but also with PD specific characteristics (e.g., PD fallers showing higher variability than older adult fallers) [27, 29]. Real-world digital outcomes show promising results to quantify novel composite indexes (e.g., combining information on falls rate with walking activity) sensitive to change in fall risk in intervention studies [30–32]. Despite the availability of real-world falls repositories [33], methodologies for real-world automatic fall detection remain challenging, prone to the detection of false positives [34], and not thoroughly addressed in PD [35].

FoG is also notoriously difficult to fully replicate and detect, increasing the potential value of remote monitoring. To elicit FoG episodes, studies have tested participants in both ON and OFF conditions during scripted tests in the lab and home and used ML models [36–38]. The sensitivity and specificity for FoG detection both increase (88.09% and 88.01% respectively) when personalized (BWS data labeled by the participant, so user-dependent) rather than generic (automatic, user-independent) models are used [36, 37]. Comparisons between freezers and non-freezers indicate that the “quality of turning” digital outcome (e.g., turning angle smaller), rather than quantity of mobility, was impaired in PD freezers [25].

While construct validity is often reported (in terms of moderate relationship with clinical scales and discriminant validity) for fall risk and FoG, criterion validity is often limited to testing in the laboratory, rather than real-world environments for falls risk. Videos and ML techniques are mainly devoted to FoG.

### Physical activity

Daily-living physical activity is one of the more mature applications of BWS. Outcomes such as the intensity of movement (e.g., energy expenditure, METs, step count) and temporal periods (bouts) of physical activity can be quantified [39]. Quantification of physical activity DMOs are based on features that describe the “magnitude” of BWS signals (e.g., counts, METs) or walking related features (based on identification of events—e.g., steps in the BWS signal for step count).

Commercial devices are widely utilized in PD for quantifying physical activity [40–42]. Although people with PD have lower levels of physical activity compared to older adults (discriminant validity), construct validity provides contrasting results with either no [42], moderate [43] or strong [44] relationships with clinical scales (e.g., UPDRS, Hoehn and Yahr staging) [40]. Criterion validity was again limited to laboratory-based tests (e.g., MET) rather than real-world environments [44], where the use of self-report diaries limit validity assessment due to subjectivity and recall issues [42].

### Current limitations

Across the clinical concepts of interests presented, the vast majority of studies use a single or combination of BWS for data logging and off-line analysis with developed analytics. Only a few examples developed connected systems (e.g., multiple sensors systems, smartphones) and online/m-health (“cloud”) platforms to achieve true remote monitoring in real-time (e.g., REMPARK, PD_Monitoring) [14, 45]. These m-health platforms have been used in small studies [45], focusing on selective aspects (e.g., ON-OFF state). Although good usability and user satisfaction results were reported, feasibility aspects (e.g., limited sensor battery time of 20 hours) for clinical adoption were not thoroughly investigated.

Generally, studies using BWS are cross-sectional, with only a few examples assessing the ability to detect change and responsiveness in longitudinal or interventional studies [30, 46]. This aspect needs to be better explored. Only when digital outcomes derived from BWS demonstrate robust criterion and construct validity and equal or superior clinimetric properties (e.g., sensitivity to change, prediction of outcomes) compared to conventional clinical outcomes will their application become widespread.

Importantly, except for the Parkinson’s Kinetigraph for motor symptoms (tremor, bradykinesia and dyskinesia) [12] and Fitbit for electrocardiogram App [40], another common characteristic across clinical concepts of interest and BWS is the lack of qualification reports accepted for a PD context of use by regulatory bodies (e.g., EMA, FDA). This absence precludes the widespread clinical adoption of BWS and their related digital outcomes [2]. Therefore, despite the promise of BWS for remote monitoring, technical, validity, and regulatory limitations remain significant barriers to their uptake.

## FUTURE PERSPECTIVES: HOW CAN WE MOVE FORWARD?

This brief review of the state-of-the-art shows that, although promising, widespread adoption of BWS in clinical settings is yet to transpire, likely because of several factors. To date, there has been no comprehensive demonstration of criterion (“technical”) and construct (“clinical”) validity, with differences in BWS and measurement techniques accounting for differences in reporting of results of the same mobility variables [7]. As highlighted earlier, the majority of algorithms that have been developed have not been validated in real-world conditions, which may be due in part to a lack of gold-standard references against which to test. Establishing technical and clinical validity, in addition to the demonstration of feasibility and usability of BWS in patients is essential to obtain qualification approval by regulatory bodies, and, as a consequence, more widespread use of BWS by clinicians [3].

Moreover, to truly transform clinical and research conventions, there needs to be sufficient evidence to show that remote monitoring is clearly “better” in some way (e.g., cost, discriminative and predictive value, clinimetric properties, healthcare economics) than traditional scales and methods. Last year, the Movement Disorder Society (MDS) Task Force on technology published concrete steps to facilitate adoption of BWS in clinical practice [3]. Nonetheless, although there are many examples and attempts in the growing literature to address selective aspects of the Task Force recommendations (e.g., validity and utility), definitive prospective and comprehensive studies are lacking. To move this field forward significantly, previous suggestions included the need to improve cross-discipline communication and larger collaborative efforts. Recent Innovative Medicines Initiative projects (e.g., Mobilise-D (https://www.mobilise-d.eu/), IDEA-FAST (https://idea-fast.eu/)) are paving the way to achieve this.

**Fig. 1:**
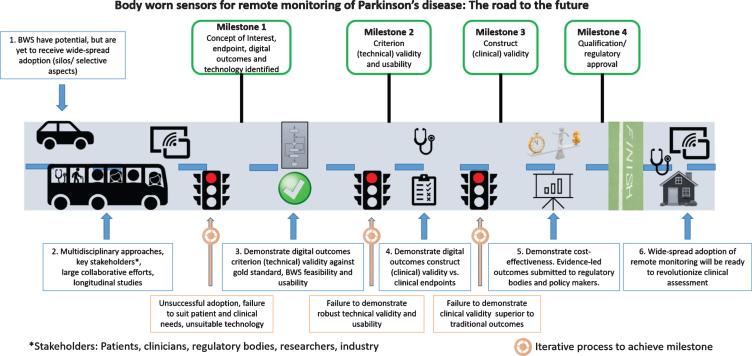
Roadmap for adoption of body worn sensors (BWS) and digital outcomes in clinical practice.

To address this need, we tried to summarize previous recommendations [2, 3, 47, 48] by proposing a roadmap with clear milestones to guide the practical clinical adoption of BWS and digital outcomes (Fig. 1):

1a. A joint effort between clinicians and end-users (e.g., patient groups [47]) should identify critical relevant clinical concepts of interest (e.g., gait), related digital outcomes (e.g., real-world walking speed), the context of use, and clinical endpoints (e.g., falls risk).
1b. Select or develop appropriate technology (BWS) with key stakeholders (industry, res-earchers, clinicians, end-users) for quantification of identified digital outcomes.
2. If not already established, demonstrate criterion (technical, e.g., cross-sectional studies) validity of the digital outcome against a gold-standard reference, in real-world conditions, feasibility, and usability of the BWS for the end-users. This could be achieved, for example, by selecting a digital outcome (e.g., walking speed) and collecting data under controlled conditions with BWS and a gold standard that can quantify the same digital outcome and that can then be used also in real-world conditions.
3. Demonstrate at least equivalent, but preferably superior construct (“clinical”, e.g., longitudinal studies) validity of the digital outcome with respect to traditional measures. This could be done, for example, by demonstrating, in a longitudinal study, that the selected digital outcome at baseline (e.g., real walking speed) has stronger correlation (or predictive power) with the clinical endpoint of interest (e.g., perspective number of falls) than clinical scales or questionnaires (e.g., UPDRS III).
4. Describe the context of use and validation work for submission to qualification/regulatory bodies for approval (EMA, FDA).
5. Demonstrate cost-effectiveness (e.g., saving time of the clinician, improving the quality of life of patients). The barrier for adoption of low-cost solutions is lower than that of solutions that require large monetary investments. This could be demonstrated by carrying out a cost-effectiveness analysis, for example, by quantifying the cost of the BWS (both in monetary and time/effort terms) versus that of a gold standard or clinical assessment and showing evidence of a lower healthcare expenditures and better outcomes achieved with BWS (e.g., as described in recommendation 3).

## CONCLUSIONS

Although BWS and digital outcomes have shown potential for clinical management, they have not yet achieved widespread clinical adoption. We can imagine a future where true remote monitoring of digital outcomes is used to enhance PD diagnosis, monitor progression, and facilitate clinical management. We hope that the recommendations and practical roadmap that are outlined in Fig. 1 will help to move the field forward toward that vision and to better care and monitoring of people with PD.

## CONFLICT OF INTEREST

JH reports having submitted a patent for assessment of mobility using wearable sensors in Parkinson’s disease. The intellectual property rights are held by the Tel Aviv Medical Center. All of the authors declare that they have no conflicts of interest.
